# A unique, newly discovered four-member protein family involved in extracellular fatty acid binding in *Yarrowia lipolytica*

**DOI:** 10.1186/s12934-022-01925-y

**Published:** 2022-10-01

**Authors:** Djamila Onésime, Léa Vidal, Stéphane Thomas, Céline Henry, Véronique Martin, Gwenaëlle André, Piotr Kubiak, Philippe Minard, Ewelina Celinska, Jean-Marc Nicaud

**Affiliations:** 1 Micalis Institute, INRAE, AgroParisTech, Université Paris-Saclay, 78350 Jouy-en-Josas, France; 2 Plateforme d’Analyse Protéomique Paris Sud-Ouest (PAPPSO), INRAE, MICALIS Institute, Université Paris-Saclay, 78350 Jouy-en-Josas, France; 3 INRAE, MaIAGE, Université Paris-Saclay, 78350 Jouy-en-Josas, France; 4 Department of Biotechnology and Food Microbiology, Poznan University of Life Sciences, ul. Wojska Polskiego 48, 60-627 Poznań, Poland; 5 CEA, CNRS, Institute for Integrative Biology of the Cell (I2BC), Université Paris-Saclay, 91198 Gif-sur-Yvette, France; 6 INRAE-AgroParisTech, UMR1319, Team BIMLip: Integrative Metabolism of Microbial Lipids, Micalis Institute, Domaine de Vilvert, 78352 Jouy-en-Josas, France

**Keywords:** *Yarrowia lipolytica*, Lipid, Fatty-acid-binding protein, Fatty-acid transport, Secretion

## Abstract

**Background:**

*Yarrowia lipolytica*, a nonconventional oleaginous yeast species, has attracted attention due to its high lipid degradation and accumulation capacities. *Y. lipolytica* is used as a chassis for the production of usual and unusual lipids and lipid derivatives. While the genes involved in the intracellular transport and activation of fatty acids in different cellular compartments have been characterized, no genes involved in fatty acid transport from the extracellular medium into the cell have been identified thus far. In this study, we identified secreted proteins involved in extracellular fatty acid binding.

**Results:**

Recent analysis of the *Y. lipolytica* secretome led to the identification of a multigene family that encodes four secreted proteins, preliminarily named UP1 to UP4. These proteins were efficiently overexpressed individually in wild-type and multideletant strain (Q4: *Δup1Δup2Δup3Δup4*) backgrounds. Phenotypic analysis demonstrated the involvement of these proteins in the binding of extracellular fatty acids. Additionally, gene deletion and overexpression prevented and promoted sensitivity to octanoic acid (C8) toxicity, respectively. The results suggested binding is dependent on aliphatic chain length and fatty acid concentration. 3D structure modeling supports the proteins’ role in fatty acid assimilation at the molecular level.

**Conclusions:**

We discovered a family of extracellular-fatty-acid-binding proteins in *Y. lipolytica* and have proposed to name its members eFbp1 to eFbp4. The exact mode of eFbps action remains to be deciphered individually and synergistically; nevertheless, it is expected that the proteins will have applications in lipid biotechnology, such as improving fatty acid production and/or bioconversion.

**Supplementary Information:**

The online version contains supplementary material available at 10.1186/s12934-022-01925-y.

## Introduction

*Yarrowia lipolytica* is a yeast species known for its high capacity for assimilation, de novo synthesis, and storage of lipids and lipid derivatives. The yeast model *Saccharomyces cerevisiae* usually displays lipid accumulation levels lower than 20% of dry weight (except some rare isolate) and that have a 1:1 triacylglycerol-to-sterol ester (TAG:SE) ratio. In contrast, *Y. lipolytica* can natively display lipid accumulation levels that exceed 50% of dry weight and that have a TAG:SE ratio of 3:1, which is highly desirable in biotechnological species. Indeed, owing to such characteristics, *Y. lipolytica* has become a model yeast species in research on lipid metabolism and turnover [1, 2].

The process of hydrophobic substrate (HS) assimilation is highly complex. It involves multiple metabolic pathways localized in different cellular compartments. Depending on environmental stimuli, the onset of the assimilation process may involve emulsification and/or enzymatic hydrolysis. Emulsification is executed by a small, extracellular glycoprotein called liposan, whose synthesis is stimulated by growth in HSs [3, 4]. While liposan’s chemical composition has not been definitively characterized, estimated ranges suggest a protein content of 5–50%, lipid content of 10–75%, and carbohydrate content of 20–83% [4–6]. Its action substantially decreases the size of lipid droplets formed in water-based media, increasing accessibility to nonmiscible HSs. Growth on TAGs requires the additional step of enzymatic hydrolysis, which is catalyzed by members of the lipase(/esterase) families [4, 7, 8]. *Y. lipolytica* genome mining revealed a gene family encoding lipases (GL3R0084) with sixteen members, including Lip2 (*YALI0A20350g*), Lip4 (*YALI0E08492g*), Lip5 (*YALI0E02640g*), and Lip7 to Lip19 (for details, see [7]). In addition, a four-member lipase/esterase family (GL3C3695) has been identified. It is composed of Lip1 (*YALI0E10659g*), Lip3 (*YALI0B08030g*), Lip6 (*YALI0C00231g*), and Lip20 (*YALI0E05995g*). The enzymes differ in substrate specificity, activity, and expression profile [4, 9–12]. In particular, it has been demonstrated that the secreted Lip2 is responsible for the vast majority of extracellular lipolytic activity. Overall, the presence of such broad multigene families reflects the high level of adaptation of this yeast to growth on HSs.

The inducible production of surfactant and lipases is part of the *surface-mediated transport* mechanism [4]. The second mechanism that enables efficient HS utilization is *direct interfacial transport*, which relies on the HS droplets binding to the cell surface [4]. This latter mechanism is mediated by an HS-inducible decrease in cell surface polarity and by the exposure of specific protrusions or hydrophobic outgrowths that collectively dock HS droplets on the cell surface [13]. These protrusions appear to be electron-dense channels that connect the exposed terminus of the protrusion with the cell interior [1, 13, 14]. Subsequently, the HS (a fatty acid [FA] or alkane) is passed through the cell membrane and is metabolized by the cell. It is either degraded via β-oxidation to produce energy, incorporated into membrane structures, or stored in specialized lipid bodies (LBs) for further use. The downstream compartments that contain enzymes involved in HS degradation are the endoplasmic reticulum, the mitochondria, the LBs, and, above all, the peroxisomes. The compartment in which an alkane or FA will be processed depends on aliphatic chain length [8, 15–17]. Long-chain alkanes and FAs are activated in the cytoplasm by fatty-acyl-CoA-synthetase (*FAA1*); they are then shuttled into the peroxisomes by the action of the transporters PXA1/PXA2. In contrast, medium- and short-chain FAs are activated not in the cytoplasm but rather in the peroxisomes, via the action of peroxisomal fatty-acyl-CoA synthetase (AAL) genes. The mode of their passage into the peroxisomes remains elusive.

There is a correlation between *Y. lipolytica*’s marked capacity for lipid assimilation, de novo synthesis, and accumulation and the broad number of genes involved in lipid turnover. Other lipid-related genes are present in addition to the aforementioned multigene family of lipases (/esterases). For example, acyl-CoA oxidase (*AOX*) catalyzes the first, rate-limiting step of peroxisomal β-oxidation. A single copy occurs in the *S. cerevisiae* genome, but the *Y. lipolytica* genome contains six Aox isoenzymes (encoded by *POX1* to *POX6* genes), each with different substrate specificities and activity levels [18, 19]. Among them, Aox2 preferentially oxidizes long-chain acyl-CoAs [20]; Aox3 specifically acts on short-chain acyl-CoAs [21]; Aox4p and Aox5p both act independently of substrate length [19]; and Aox1 and Aox6 specifically carry out dicarboxylic acid degradation [22]. Moreover, the *Y. lipolytica* genome surveys revealed that, for the transformation of alkene to alcohol, there is a single gene coding for NADPH-cytochrome P450 reductase, but there are as many as 12 genes coding for cytochrome P450 isoforms. The multigene family of cytochrome P450s (*ALK* genes) includes *ALK1*, *ALK5*, and *ALK11,* which encode enzymes that specifically handle short-chain alkanes (C10), and *ALK2*, which specifically deals with long-chain alkanes (C16) [23, 24]. There is also evidence suggesting that *ALK3*, *ALK5*, and *ALK7* encode enzymes with specificities for short-chain FAs and that *ALK2*, *ALK5*, *ALK7*, and *ALK10* encode enzymes with specificities for long-chain FAs [1, 2, 4, 11]. Correspondingly, transcription factors that activate the expression of alkane-inducible genes belong to a three-member family of proteins, Yas1 to Yas3; they bind to the alkane-responsive element (ARE1) [25–27]. Likewise, in *S. cerevisiae*, a single fatty-acyl-CoA-synthetase (ScFaa2) catalyzes the cytoplasmic activation of FAs prior to their oxidation in the peroxisomes, but as many as 10 genes encoding Aal isozymes have been identified in *Y. lipolytica* [28]. Eight of the Aals were upregulated by HSs in the medium, and all 10 contained the peroxisomal targeting signal PTS (SKL). Complementation tests conducted in the *faa1Δant1Δ* background (Ant1 is a peroxisomal ATP transporter; [15, 16]) showed that overexpression of the cytoplasmic version of Aal1 partly restored the growth of the mutant on media containing short-, medium-, and long-chain FAs, while overexpression of Aal2 to Aal10 enabled growth only on media containing short-chain FAs. Additional research indicated that Aal4 and Aal6 present substrate specificities for C16:1 and/or C18:0 [28]. Collectively, this work demonstrates the complexity and multiplicity of the genes involved in lipid metabolism in *Y. lipolytica*.

While much is known about lipid metabolism in *Y. lipolytica*, specific details remain scarce about the aliphatic moiety’s passage through the plasma membrane. Early studies on the uptake kinetics of different FAs provided the first insights into this process [29]. It was suggested that FAs were internalized in *Y. lipolytica* cells via a saturable, carrier-mediated mechanism that was substrate concentration dependent but energy independent, given that the process operated irrespective of metabolic energy levels or membrane potential formation. It was also suggested that at least two individual transportation systems with different specificities co-exist in *Y. lipolytica*. Competition experiments clearly demonstrated that one system specifically acted on C12-C14, while the other specifically acted on C16-C18. It was also shown that *Y. lipolytica* is unable to internalize C8 or C10, which, in addition to the high toxicity of short-chain FAs and alkanes [30, 31], is the reason for its inability to efficiently grow on these substrates. Other studies have suggested that transporters belonging to the ABC1 family may play a role in moving alkenes through the plasma membrane [22]. Four genes highly homologous to the *ABC1* gene were identified in the *Y. lipolytica* genome: *ABC1* (*YALI0E14729g*), *ABC2* (*YALI0C20265g*), *ABC3* (*YALI0B02544g*), and *ABC4* (*YALI0B12980g*). Previous research suggested that Abc1 and Abc2 may be involved in the transportation of C16 and C10 alkanes, respectively. Another study showed that, in *Y. lipolytica*, the transcription of the Abc2 and Abc3 transporters was enhanced upon exposure to a range of alkanes [31]. Overexpression experiments in *S. cerevisiae* revealed that Abc2 and Abc3 act as efflux pumps, leading to improved host tolerance of C9 and C10 alkanes. In addition, Fat1 (*YALI0E16016g*) acts as a very-long-chain-fatty-acyl-CoA synthetase and was suggested to also be involved in FA transportation across the cell membrane [17]. That said, the exact mechanism by which hydrophobic compounds pass through a membrane remains in dispute, and the genes involved have yet to be identified. Systematic screening of insertional mutants [15, 32, 33] has yet to allow the identification of the individual genes concerned. Therefore, we used information from previous systematic insertional mutant screening and knowledge on other multigene families involved in lipid metabolism in *Y. lipolytica* to arrive at the hypothesis that the genes involved in FA/alkane internalization belong to a multigene family that encodes proteins with overlapping specificities.

In this study, we used secretory proteome data mining to identify a unique protein family composed of four members, which we have preliminarily named UP1 to UP4 (UP = unknown protein). The sequences of these proteins bear no similarities to the sequences of any other known proteins. Further examination of this hereto uncharacterized gene family led us to hypothesize that its members could be involved in FA fixation. After phenotypically characterizing a quadruple-deletion mutant and individual overexpression strains, we concluded that the biological function of the family’s members is FA internalization. The growth profiles of the constructed strains suggested that the family’s members possess overlapping substrate specificities for aliphatic chain length. Bioinformatic 3D modeling of the proteins confirmed their structural adaptation to FA binding. Altogether, these results highlight the existence of a unique, previously undescribed gene family in *Y. lipolytica* that encodes FA-binding proteins. The proposed name for the newly discovered protein family is eFbp (for extracellular-fatty-acid-binding protein).

## Results

### Newly identified protein family found in the *Y. lipolytica* secretome—basic analysis of amino acid sequences

Recent work performed high-throughput proteomics on the total secretome of the *Y. lipolytica* W29 strains used to produce heterologous proteins under industrial fermentation conditions (10-L fermenters in fed-batch mode) (Onésime et al., to be published). Secretome data mining conducted with X!TandemPipeline allowed the identification of three proteins of unknown function, encoded by *YALI0C05687g*, *YALI0D03245g*, and *YALI0F04620g*. Respectively, coverage was 7.08% (7.66%), 11.66% (12.62%), and 19.03% (20.57%) for the complete form (and the mature form), and the E-values were 50.199, 62.872, and 23.919. Blasting the *Y. lipolytica* genome against the GRYC database showed that these proteins are encoded by a multigene family of four members (fourth member = *YALI0F04598g*) (Table 1). Sequence-based predictions indicated that the polypeptide chains are composed of 223–226 amino acids (complete form)/206–209 amino acids (mature form); have a molecular weight ranging from 21.678 to 22.725 kDa; and display an isoelectric point (pI) between 5.1 and 8.1. The systematic gene names taken from the genomes of *Y. lipolytica* strains E150 and W29 and the abbreviated versions of these names used hereafter are given in Table 1.

**Table 1:** Nomenclature and basic biochemical characteristics of the newly identified proteins

E150 strain reference genome	W29 strain reference genome	Working name	Proposed new name	AA number*	Molecular weight* [Da]	Predicted pI*
YALI0D03245g	YALI1_D04128g	UP1	eFbp1	223 (206)	21,717.43	5.40
YALI0F04598g	YALI1_F07042g	UP2	eFbp2	224 (207)	21,678.16	5.10
YALI0C05687g	YALI1_C07288g	UP3	eFbp3	226 (209)	21,950.50	6.47
YALI0F04620g	YALI1_F07093g	UP4	eFbp4	226 (209)	22,725.72	8.09

Systematic gene names from the E150 and W29 wild-type strain genomes. Number of amino acids in the polypeptide chain, predicted molecular weight, and predicted isoelectric point (pI) of the mature form. The molecular weight prediction was based on the mature form and is expressed in Da (monoisotopic mass from Expasy)

*AA* amino acid

^*^For the mature form

**Fig. 1:**
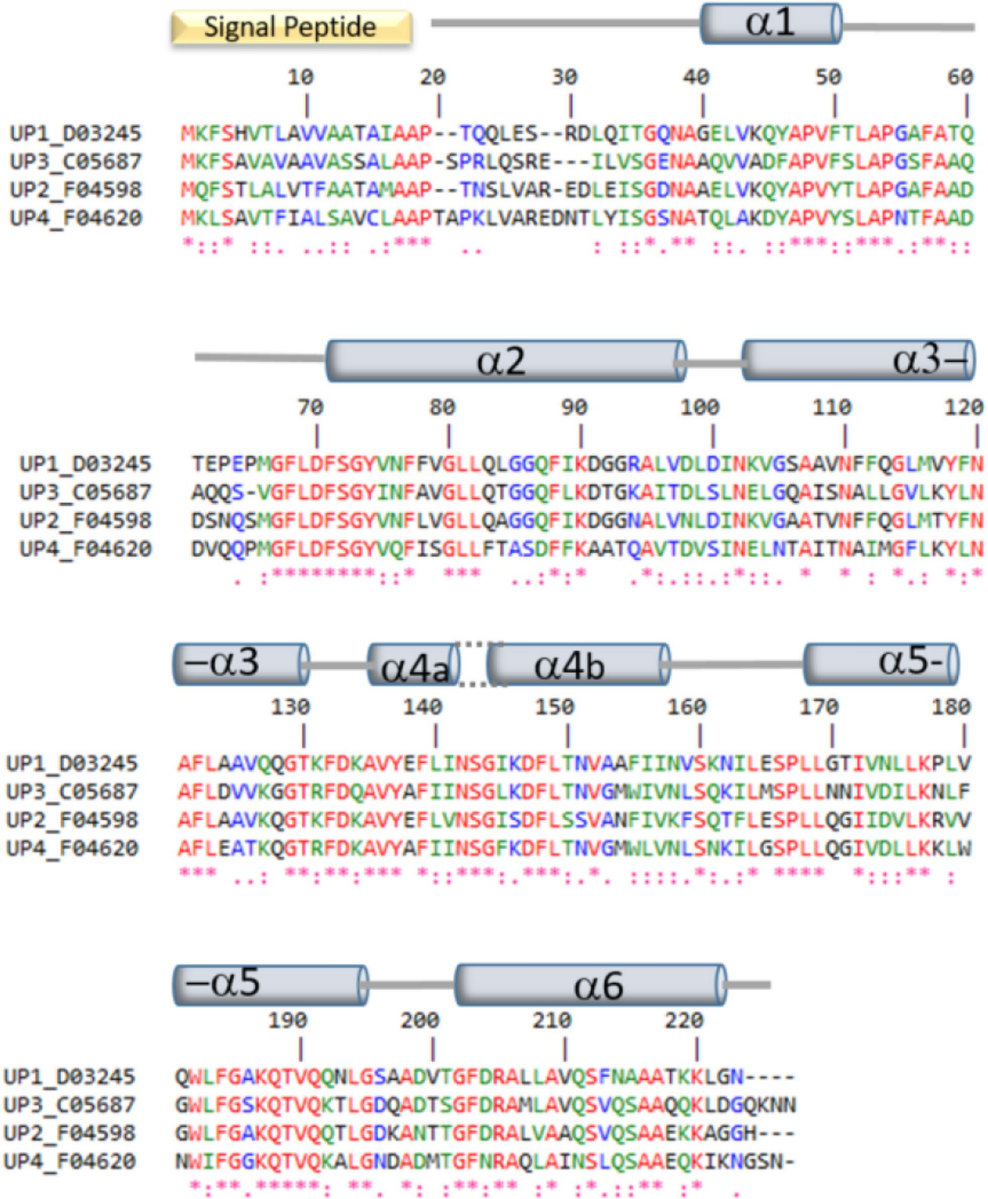
Multiple sequence alignment of the four unknown proteins—UP1 to UP4. The predicted signal peptide is highlighted by the yellow box above the sequences. Identical amino acids, conserved amino acids, and similar amino acids are indicated in red, blue, and green, respectively. The systematic gene names were abbreviated using the chromosome letter and the gene number (e.g., YALI0D03245g was abbreviated as D03245g). The position and nature of the secondary structural elements predicted by alpha-fold 3D structure prediction (for UP1) are indicated by light gray cylinders above the sequences. The structure of helix α4 is locally distorted; for this reason, it was split into two parts (α4a and α4b)

Comparison of the amino acid sequences showed that the four proteins are highly similar (50–70% sequence identity; Fig. 1). In addition, all the proteins have a predicted 17-amino-acid signal peptide, with a probability of 0.9946 for D03245 (UP1) (MKFSHVTLAVVAATAIA), 0.9991 for F04598g (UP2) (MQFSTLALVTFAATAMA), 0.9926 for C05687g (UP3) (MKFSAVAVAAVASSALA), and 0.9995 for F04620g (UP4) (MKLSAVTFIALSAVCLA). For each protein, a similar 3D folding pattern composed of a six-helices bundle was predicted (Fig. 1 and section on 3D structure modeling).

### Uniqueness of UP sequences due to lack of similarity with other protein sequences

The complete UP sequences were subsequently blasted against available protein sequence databases. Strikingly, the only similarity was found with homologous sequences from other *Y. lipolytica* strains (apart from E150 and W29), such as German H222 and Polish A101 (data not shown). Since this screening process failed to yield any significant hits beyond the *Y. lipolytica* homologs, a search was performed using the conserved stretches of amino acid sequences found during the multiple alignment. Seven conserved motifs, numbered from 1 to 7, were identified within each UP: AAP[TS], APV[FY][TS]LAPxxFA, GFLDFSGY, GT[KR]FD[KQ]AVY[EA]F[IL][VI]NSGx[KS]DFL, [IF]LxSPLL, W[IL]FGxKQTVQ, and [TS]GF[DN]RA. They served as queries in searches against the NCBI and UniProt protein sequence libraries. As expected, motif 1, localized at the signal peptide (at the C-terminus), was present in the largest number of protein sequences stored in the NCBI and UniProt databases (3,192,524 and 2,106,782 hits, respectively). Similarly, motifs 5 and 7 were relatively frequently found (18,712/13174 and 7511/5086 hits). In contrast, motifs 2, 4, and 6 were identified in only five (NCBI) or seven (UniProt) protein sequences. Strikingly, irrespective of the database queried, the four UPs were among the motif-bearing proteins identified. This result highlights that each of these conserved motifs is always accompanied by the other two and that the motifs are specific and unique to the newly identified protein family.

### Cloning and overexpression of the UP genes

Since the biological function of the UPs could not be inferred from the searches of the protein databases, overexpression strains were designed and constructed. Each UP gene was amplified using a specific primer pair (Additional file 1: Table S1) from W29 wild-type genomic DNA and was cloned as a BamH1/AvrII fragment into JMP4230 (Additional file 1: Fig. S1), giving rise to the plasmids JMP4440, JMP4442, JMP4444, and JMP4448 for UP1, UP2, UP3, and UP4 overexpression, respectively (Table 2). The individual genes were overexpressed under the control of a strong erythritol-inducible promoter [34].

**Table 2:** Plasmids used in this study

Plasmid name	Characteristics	Use	References
JMP4230	JMP62 pHu8EYK URA3ex	Overexpression	[35]
JMP4440	UP1; D03245g cloned in JMP4230	Overexpression	This study
JMP4442	UP2; F04598g cloned in JMP4230	Overexpression	This study
JMP4444	UP3; C05687g cloned in JMP4230	Overexpression	This study
JMP4448	UP4; F04620g cloned in JMP4230	Overexpression	This study
JMP4472	GGA-URA3ex_CRISPRrCas9-yl_RFP	gRNA for gene disruption	[36]
JMP4393	GGA-LYS5ex_CRISPRrCas9-yl_RFP	gRNA for gene disruption	[36]

The expression cassettes were liberated from the plasmid by NotI digestion and transformed in strain JMY7126, which is a deletion mutant without any of the three genes encoding the main secreted lipases (Lip2, Lip7 and Lip8) or the *EYK1* gene for optimal erythritol induction [35]. This cloning strategy gave rise to strains JMY7283 (overexpressing UP1), JMY7287 (overexpressing UP2), JMY7291 (overexpressing UP3), and JMY7295 (overexpressing UP4) (Table 3).

**Table 3:** Strains used in this study

Name	Genotype	Auxotrophy	References
JMY399	French W29 wild-type strain	No	[3]
JMY7126	*MATA ura3*-*302 leu2*-*270*-*LEU2*-*Zeta*, *xpr2*-*322*, *lip2Δ*, *lip7Δ*, *lip8Δ*, *lys5Δ*, *eyk1Δ*	Ura^−^, Lys^−^	[35]
JMY7283	JMY7126 + jmp4440 **D03245g** (UP^oe^)	Lys^−^	This study
JMY7287	JMY7126 + jmp4442 **F04598g** (UP2^oe^)	Lys^−^	This study
JMY7291	JMY7126 + jmp4444 **C05687g** (UP3^oe^)	Lys^−^	This study
JMY7295	JMY7126 + jmp4446 **F04620g** (UP4^oe^)	Lys^−^	This study
JMY8651	JMY7126 + *GGE114 + **YALI0B21582gΔ** (fil-; *mhy1Δ*)	Lys^−^	This study
JMY8673	JMY7126 + **YalI0C05687g*Δ**** (Q1-up3Δ)*	Ura^−^, Lys^−^	This study
JMY8674	JMY7126 + **YalI0D03245g*Δ**** (Q1-up1Δ)*	Ura^−^, Lys^−^	This study
JMY8675	JMY7126 + **YalI0F04598g*Δ**** (Q1-up2Δ)*	Ura^−^, Lys^−^	This study
JMY8683	JMY7126 + **YalI0F04620g*Δ**** (Q1-up4Δ)*	Ura^−^, Lys^−^	This study
JMY8684	JMY8674 + **YALI0F04598gΔ** (Q2-*up1Δ up2Δ*)	Ura^−^, Lys^−^	This study
JMY8700	JMY8684 + **YALI0F04620gΔ** (Q3-*up1Δ up2Δ up4Δ*)	Ura^−^, Lys^−^	This study
JMY8748	JMY 8700 + **YALI0C05687gΔ** (Q4-*up1Δ up2Δ up4Δ up3Δ*)	Ura^−^, Lys^−^	This study
JMY8761	JMY8748 + **YALI0B21582gΔ** (Q4, fil-; Q4 *mhy1Δ*)	Ura^−^, Lys^−^	This study
JMY8777	JMY8761 + jmp4230 (Q4 URA3)	Lys^−^	This study
JMY8778	JMY8761 + jmp4230 (Q4 URA3)	Lys^−^	This study
JMY8779	JMY8761 + jmp4444 **C05687g** (Q4-*mhy1*Δ UP3^oe^)	Lys^−^	This study
JMY8780	JMY8761 + jmp4444 **C05687g** (Q4-*mhy1*Δ UP3^oe^)	Lys^−^	This study
JMY8781	JMY8761 + jmp4440 **D03245g** (Q4-*mhy1*Δ UP1^oe^)	Lys^−^	This study
JMY8782	JMY8761 + jmp4440 **D03245g** (Q4-*mhy1*Δ UP1^oe^)	Lys^−^	This study
JMY8783	JMY8761 + jmp4442 **F04598g** (Q4-*mhy1*Δ UP2^oe^)	Lys^−^	This study
JMY8784	JMY8761 + jmp4442 **F04598g** (Q4-*mhy1*Δ UP2^oe^)	Lys^−^	This study
JMY8785	JMY8761 + jmp4446 **F04620g** (Q4-*mhy1*Δ UP4^oe^)	Lys^−^	This study
JMY8786	JMY8761 + jmp4446 **F04620g** (Q4-*mhy1*Δ UP4^oe^)	Lys^−^	This study

*oe* overexpression

### Synthesis and secretion of UPs by erythritol-inducible strains

**Fig. 2:**
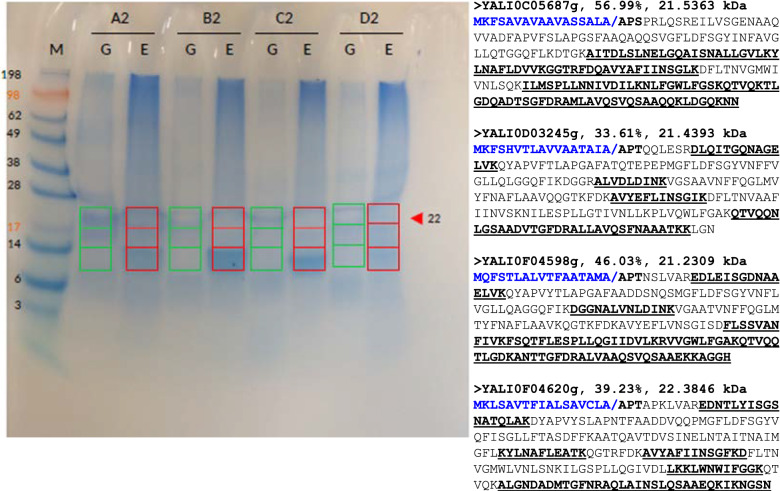
Analysis of UP synthesis and secretion by the overexpression strains. Left: SDS–PAGE separation of concentrated supernatants taken from batch cultures of the overexpression strains. Lanes A2: strain overexpressing YALI0D03245g/UP1, B2: strain overexpressing YALI0F04598g/UP2, C2: strain overexpressing YALI0C05687g/UP3, D2: strain overexpressing YALIF04620g/UP4; Lanes G: cultivation in noninduced glucose-based medium, YNBD2, and E: cultivation in induced glucose-based medium (i.e., in the presence of erythritol), YNBD2E. The molecular mass (MM) standard was SeeBlue^®^ Plus2 Pre-Stained Standard (Thermo Fisher Scientific, Villebon sur Yvette, France), which covered polypeptides from 3 to 198 kDa. The red arrow indicates the expected migration distance of the 22-kDa protein. The areas in green (non induced) and red (induced) were cut for proteomic analysis. Right; UPs amino acid sequences with peptides found by proteomic underlined. Percentage of coverage and molecular weight of the mature form are indicated

The overexpression strains were cultured using shake-flask batch cultivation. UP overproduction patterns were studied using proteomics analysis following culturing in noninduced media (YNBD2) and induced media (YNBD2E) (Fig. 2). The band patterns observed in the SDS–PAGE gels were unexpected: the most intense bands were from the erythritol-induced cultures, and they migrated down below the anticipated area (red arrow in Fig. 2).

Thus, three regions were excised from each lane—region 1, which was around the expected size of the UP proteins (approximately 22 kDa), and regions 2 and 3, with the intense bands (boxes in Fig. 2). This material underwent proteomics analysis. The aim was to determine the number of identifiable peptides in each of the bands and percent coverage under both noninduced (G) and induced (E) conditions. As shown in Table 4, the numbers of identifiable peptides were higher in the bands formed by the concentrated supernatants from the induced cultures (E). However, the UPs migrated in a pattern (< 14 kDa) that was inconsistent with their expected size. The sequence coverage for the peptides identified under induced conditions was high, ranging from 33.6 to 57% for the mature forms. Abundance was variable among UPs, as shown by the number of identifiable peptides detected under induced conditions: 11 to 19 spectra (Table 4).

**Table 4:** Proteomics analysis of secreted UPs

UP genes	A2G	A2E	B2G	B2E	C2G	C2E	D2G	D2E
UP1 D03245g	5	**17**	5	5	4	7	4	4
UP2 F04598g	2	3	2	**66**	8	3	3	2
UP3 C05687g	1	2	9	3	1	**111**	6	2
UP4 F04620g	2	5	3	4	0	10	19	**32**

The number of identifiable peptides for the respective UPs in the most intense bands (migration: < 14 kDa). Bolded UPs specific peptide number in the corresponding overexpression strains

The bands were excised from the SDS–PAGE gel (see Fig. 2) and analyzed using high-resolution mass spectrometry

Codes:* A2* concentrated supernatant from UP3 C05687g; *B2* UP1 D03245g; *C2* UP2 F04598g; and *D2* UP4 F04620g under noninduced (G) and induced (E) conditions

The proteomics analysis confirmed the proper synthesis and secretion of the UPs by the overexpression strains; levels were greatly increased under induced conditions. The analysis also indicated that nontarget UPs were also constitutively expressed from their native promoter in these media (identified at lower abundance, based on typically 1–10 identifying peptides). Such an outcome could affect the adequacy of the phenotypic analysis. Therefore, it was necessary to first construct a quadruple-deletant strain (Q4) and then construct derivatives that overexpressed individual UPs in the Q4 background.

### Overexpression of UPs in a quadruple-deletant strain (Q4-*mhy1*Δ) and phenotypic analysis

**Fig. 3:**
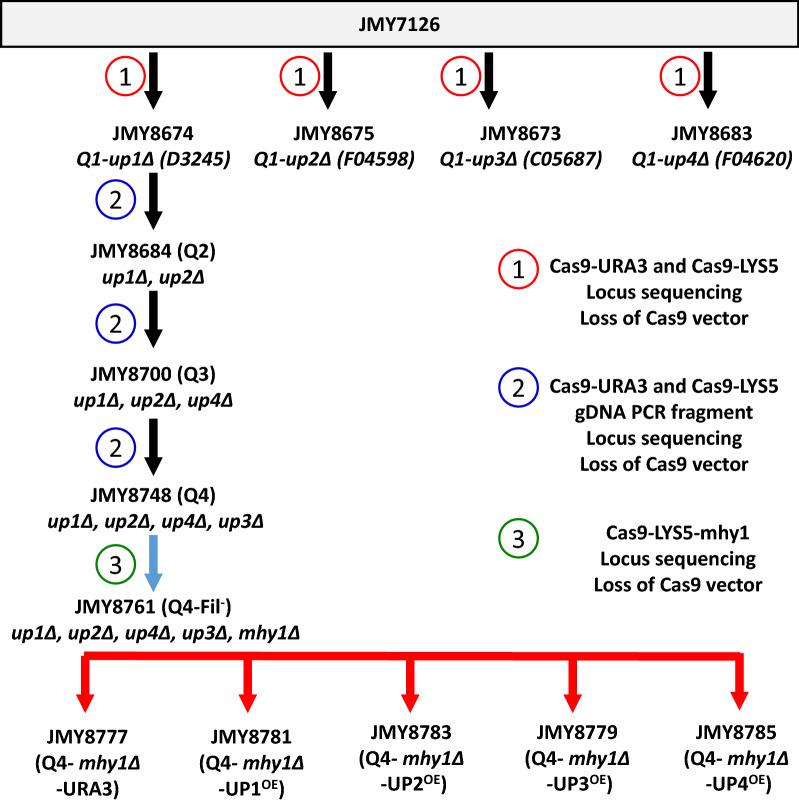
Overview of strain construction. Construction of quadruple-deletion mutant Q4 and the derivative UP-overexpression strains. Individual UP genes were deleted in the auxotrophic strain JMY7126 [35] via the CRISPR-Cas9 method and using the corresponding gRNA vectors. Further successive gene disruption was performed in the Q1 strain. Co-transformation was performed using the corresponding gRNA vectors together with the corresponding amplified genomic locus carrying the deletion of the up2Δ, up4Δ, and up3Δ loci. The *MHY1* gene was deleted prior to the introduction of the cassettes encoding the UP genes individually

Since the accuracy of the phenotypic analysis could be diminished due to the unintentional co-secretion of the non-target UPs by the overexpressing strains, a quadruple-deletant strain (Q4) was constructed. The strategy comprised successive gene deletions using the CRISPR-Cas9 method [36], as illustrated in Fig. 3. First, the replicative plasmids CRISPR-Cas9-gRNA-UPs-*URA3* and CRISPR-Cas9-gRNA-UPs-*LYS5* were constructed using the gRNA primer pair designed for the corresponding target sites (Additional file 1: Table S1). The plasmids were co-transformed into the JMY7126 strain, and prototrophic transformants were selected on minimal medium, YNBD2. After transformant selection, the corresponding UP locus was amplified, screened for deletion, and sequenced. After gene deletion had been confirmed, the strains were grown in YPD to cure the replicative CRISPR-Cas9 plasmid. Strains bearing the expected deletion were retained (Table 3). The UP1 to UP4 single-deletion mutants (Q1) were assigned the names JMY8674 (*up1*Δ), JMY8675 (*up2*Δ), JMY8673 (*up3*Δ), and JMY8683 (*up4*Δ), respectively (Fig. 3). Then, multiple gene deletion was initiated using Q1-*up1*Δ and co-transformation with the CRISPR-Cas9-gRNA-UP plasmids together with a PCR fragment amplified from the corresponding deletion strain, resulting in the Q4 strain JMY8748. In addition, since filamentation is known to affect HS phenotypic analysis, the *MHY1* gene deletion, previously shown to abolish hyphae formation [37], was also introduced into the Q4 deletion mutant using a CRISPR-Cas9-gRNA-MHY1-LYS5 vector. The resulting strain was then transformed with the UP-overexpression cassettes, resulting in the overexpression strains Q4-*mhy1*Δ-UP1^OE^, Q4-*mhy1*Δ-UP2^OE^, Q4-*mhy1*Δ-UP3^OE^, and Q4-*mhy1*Δ-UP4^OE^. Strain JMY8761 was transformed using an empty vector containing *URA3*, giving rise to JMY8777 (Q4-*mhy1*Δ-URA3), which was used as a control (Fig. 3).

### Phenotypic analysis of strain growth on HSs with different aliphatic chain lengths

**Fig. 4:**
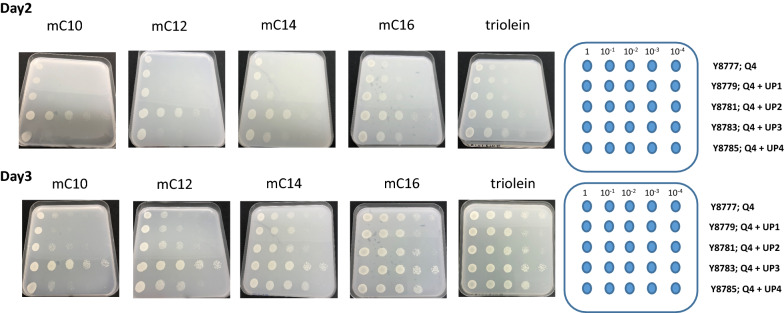
Growth of mutant strains on fatty acids. Drop tests were conducted on the methyl esters of fatty acids with different lengths of aliphatic chains (mC10 to mC16 and triolein). The quadruple-deletant Q4 (Q4-mhy1Δ-URA3) and the derivative overexpression strains Q4 + UP1 (Q4-mhy1Δ-UP1^OE)^ to Q4 + UP4 (Q4-mhy1Δ-UP4^OE^), were decimally diluted and spotted (10 μL) on minimal media containing 0.4% of the corresponding HS. Plates were incubated at 28 °C. Pictures were taken after 48 and 72 h (day 2 and 3, respectively). The drop test was conducted twice, using two subclones of each specific genotype

Assuming UP involvement in HS utilization, we characterized the growth of the quadruple-deletant strain (Q4), with all four loci knocked out, and the overexpression strains on solid plated media containing HSs of different aliphatic chain lengths. Strain JMY8777, a derivative of strain JMY8761 (transformed with the empty vector), was used as a control (Fig. 4).

As depicted in Fig. 4, growth inhibition was observed for all the strains when grown on short-chain FAs (mC10 to mC14), except for the strain overexpressing UP3, for which growth was still observed up to the 10^–3^ dilution. In contrast, on media containing longer-chain FAs (mC16 and C18:1 [triolein]), growth was observed up to the 10^–3^ dilution. This result demonstrates that the deletion of all four UPs (Q4 strain) abolishes growth on short-chain FAs, particularly on mC10, which implies they are specifically involved in short-chain FA fixation and internalization. As growth of Q4 was impaired neither on mC16 nor on triolein, these HS must be fixed and internalized by some other mechanism. Furthermore, sole overexpression of UP3 alleviated the growth inhibition particularly on mC10 but also on mC12 and mC14. This finding clearly indicates that UP3 is implicated in the transport of short-chain FAs, which was particularly obvious for mC10. Both UP2 and UP4 overexpression appeared to slightly alleviate the growth inhibition on mC12 and mC14, suggesting their specificities for these FAs. Overexpression of UP1 had a minor positive impact on strain growth, which was mainly observed on mC12, where the strain grew up to the 10^–2^ dilution vs. 10^–1^ for Q4. Based on these results, it was postulated that the UPs are involved in short- and medium-chain FA fixation and internalization. It is suggested that their operation is based on FA chain length (UP3 is the sole UP to act on mC10; UP2 and UP4 mainly act on mC12 and mC14) but with overlapping specificity (UP1 acts on mC10 to mC14). Interestingly, these patterns are consistent with sequence alignment, which ranges from UP1 to UP3 to UP2 and UP4.

### Octanoic acid toxicity in Q4 and the overexpressing strains

**Fig. 5:**
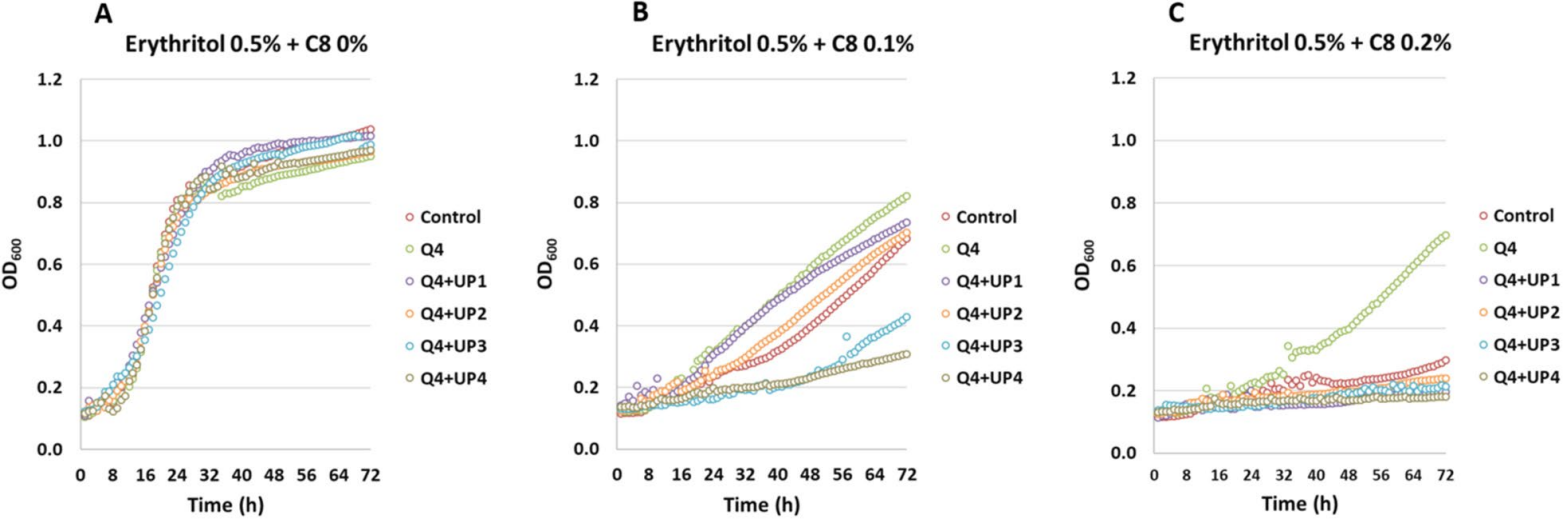
Effect of octanoic acid on the growth of the quadruple-deletant mutant (Q4) and the four derivative overexpressing strains. Growth of Q4 (Q4-mhy1Δ-URA3) and the overexpression strains, Q4 + UP1 (Q4-mhy1Δ-UP1OE) to Q4 + UP4 (Q4-mhy1Δ-UP4OE), in the presence of different concentrations of octanoic acid (C8) at 28 °C and 180 rpm. The strain JMY8651 (fil-; mhy1Δ) was used as a control. **A**: 0% C8; **B**: 0.1% C8 and **C**: 0.2% C8

Octanoic acid (C8) is known to be very toxic to *Y. lipolytica* [29, 30]. Assuming the involvement of the UPs in FA transportation (based on the drop test data; Fig. 4), we aimed to investigate the effects of C8 toxicity on the Q4 and UPs individual overexpression on C8 toxicity. All five strains were grown in minimal media supplemented with different concentrations of C8 (0% to 0.2%; Fig. 5). No major differences in growth could be observed in the absence of C8 (Fig. 5A). Growth was also monitored in the absence and presence of an inducer (erythritol). As shown in Fig. 5B, C, deletion of the four UP genes (Q4 strain) increased C8 tolerance under conditions of erythritol induction (compared to the control strain, JMY8651; vs. all the other strains: p < 0.05). Overexpression of UP3 and UP4 led to increased toxicity at 0.1% C8 (Fig. 5B), while overexpression of all the UPs caused increased toxicity at 0.2% C8 (Fig. 5C; vs. all the other strains: p < 0.05). Based on these observations, we postulate that UPs are involved in short-chain FA internalization. Additionally, signs of the substrate specificities of the UPs can be inferred from this assay, as UP3 and UP4 both showed a greater affinity for C8.

### 3D structural modeling

**Fig. 6:**
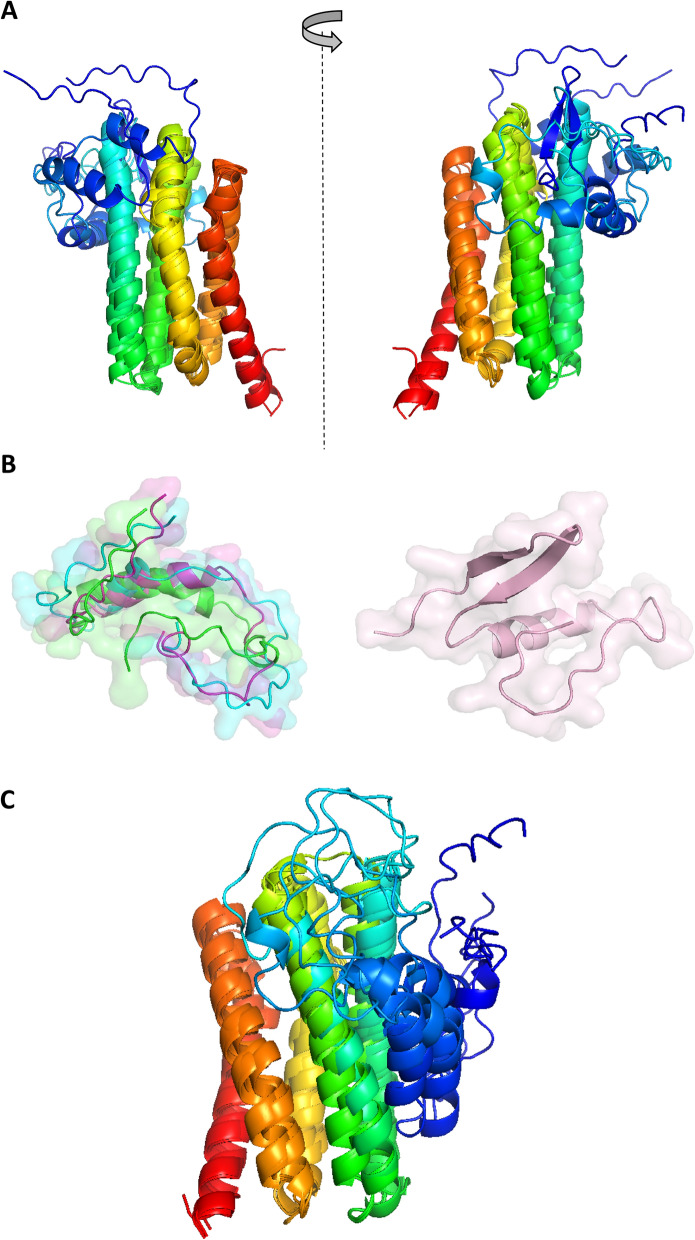
Models of the predicted 3D structures of UP1 to UP4. **A** The rank 1 models for each protein, as predicted by AlphaFold, were superimposed. Each polypeptide chain is colored blue to red from the N-terminus to the C-terminus. The two views show the opposite faces of the same structures. The five long helices form a helice bundle in the four proteins; however, the position of the N-terminus extension (in blue) was not reliably defined. **B** The N-terminus parts 1 (residues 1–50) (green), 2 (cyan), and 3 (purple) of UP1 were predicted to have similar structures, but the prediction of their relative positions with respect to the helical domain was more variable (see blue in **A**). The N-terminal part of UP4 (right) was predicted to have a single helix, similar to UP1, UP2, and UP3: a short pair of two strands that associate to form a β sheet. **C** The rank 1 to rank 5 models for UP1 that were independently predicted by AlphaFold were superimposed. Each chain is colored from blue to red from the N-terminus to the C-terminus. The five long helices (residues 50–200) are consistently predicted to occur in the same relative positions. The predicted structures of residues 1–50 were more variable

The sequences of UP1 to UP4 are nearly 40% similar in amino acid residue identity and are hence clearly homologous. Therefore, they are expected to fold into similar 3D structures. The similarity of the primary structures of UP1 to UP4 is too low with respect to the known proteins in the protein database, which makes consistent homology modeling impossible. We therefore ran the sequences of UP1 to UP4 through the AlphaFold2 computational tool. AlphaFold is a tools for family structure prediction that uses deep learning to produce high-quality structure predictions via a blind test (CASP14); it can also be used when no clear homologs are known [38]. The 3D structures of UP1 to UP4, as modeled by AlphaFold, are highly similar to each other (Fig. 6).

Their 3D structures are highly similar from position 70 (position 50 in the mature form) up to position 221, at the end of the sequence (UP1 numbering) (Fig. 6A). Consequently, the cores of UP1 to UP4 were predicted to fold into a single domain composed of five helices 30 amino acid residues long (on average) that assemble into a helix bundle (helices 2–6 in Fig. 1). Notably, helix 4 is locally disordered at the same position in all the models, centered on the conserved sequence F[IL][VI]NSGx[KS]DFL. Such results in a topological kink that could help pack the helical-bundle. The helical-bundle was expected to form an inner cavity, observed here for the four proteins. Interestingly, the N-terminus (20–70 in Fig. 1, or 1–50 in the mature form) has an extension beyond the main helical-bundle. The predicted structure for this N-terminus part is roughly similar (Fig. 6B) for UP1 to UP3 with a single α helix, but  the position of this N-terminus part of the protein relative to the main helical domain was not accurately predicted, possibly due to structural flexibility at this end. For UP4, the N-terminus region could also be predicted. In the rank 1 model, the N-terminus formed a single α helix with an additional β sheet of two strands. However, the rank 2 model did not predict these β strands but, instead, a structure very similar to the N-termini of UP1 to UP3. AlphaFold predictions of the helical domain in UP1 to UP4 appear to be reliable (Fig. 6C) based on the following criteria. First, the predicted local difference test (PLLDT) score is a per-residue confidence metric calculated by AlphaFold. The score for the rank 1 model was in the zone of 60–80 (scale: 0–100) for the main helical domain, which suggests that the overall folding pattern of this domain is highly probable. However, the structure of the N-terminus and its relative position with respect to the helical domain was less confidently predicted. Second, the predicted structures for UP1 to UP4 were highly similar to those expected for these clearly homologous proteins. Third, the independent predictions of these same sequences consistently arrived at the helical domain (e.g., see rank 1 to 5 models of UP1 in Additional file 1: Fig. S2).

### Search for known proteins with related 3D structures

The coordinates obtained from the UP1 rank 1 model were submitted to a systematic comparison against Dali’s full protein data bank (PDB) [39]. We obtained a list of nonredundant structures found to be structurally similar to UP1, as shown in Table 5. The closest structures are ligand domains 1 and 2 of the Mp1 protein in *Talaromyces marneffei* (previously known as *Penicillium marneffei*) and the ligand-binding domain of a protein in *Aspergillus fumigatus* (root-mean-square deviation of 2.6 and 2.7 Å, respectively).

**Table 5:** Relevant protein structures similar to UP1 detected via Dali

PDB	Z	Rmsd	D lali	nres	%id	Species	Protein and domain	Bound molecules
**5fb7-B**	15.4	2.7	143	151	10	*Talaromyces marneffei*	MP1 ligand-binding domain 1	Arachidonic acid (2 molecules)
**5csd-A**	15.2	3.2	147	158	10	*Talaromyces marneffei*	MP1 ligand-binding domain 2	Arachidonic acid
**5j5K-A**	14.8	2.6	142	151	11	*Aspergilus fumigatus*	AFMP4P ligand-binding domain	Palmitic acid
5csd-D	15.1	3.3	151	159	10	*Talaromyces marneffei*	MP1 ligand-binding domain 2	Arachidonic acid (2 molecules)
5ecf-B	15.3	2.8	142	150	9	*Talaromyces marneffei*	MP1 ligand-binding domain 1	Arachidonic acid
5e7x-A	15.2	3.1	147	155	9	*Talaromyces marneffei*	MP1 ligand-binding domain 1	Palmitic acid
6zpp-A	13.4	2.9	144	157	7	*Drechmania coniospora*	Virulence factor	

*rmsd* root-mean-square deviation of the equivalent Cα between the UP1 model and a protein from the PDB found to be structurally similar; *D lali* length of the structural alignment; *nres* number of residues = length of chain; *%id* % identity between the two proteins

Z-score as calculated by Dali, where a score above 2 is considered significant

**Fig. 7:**
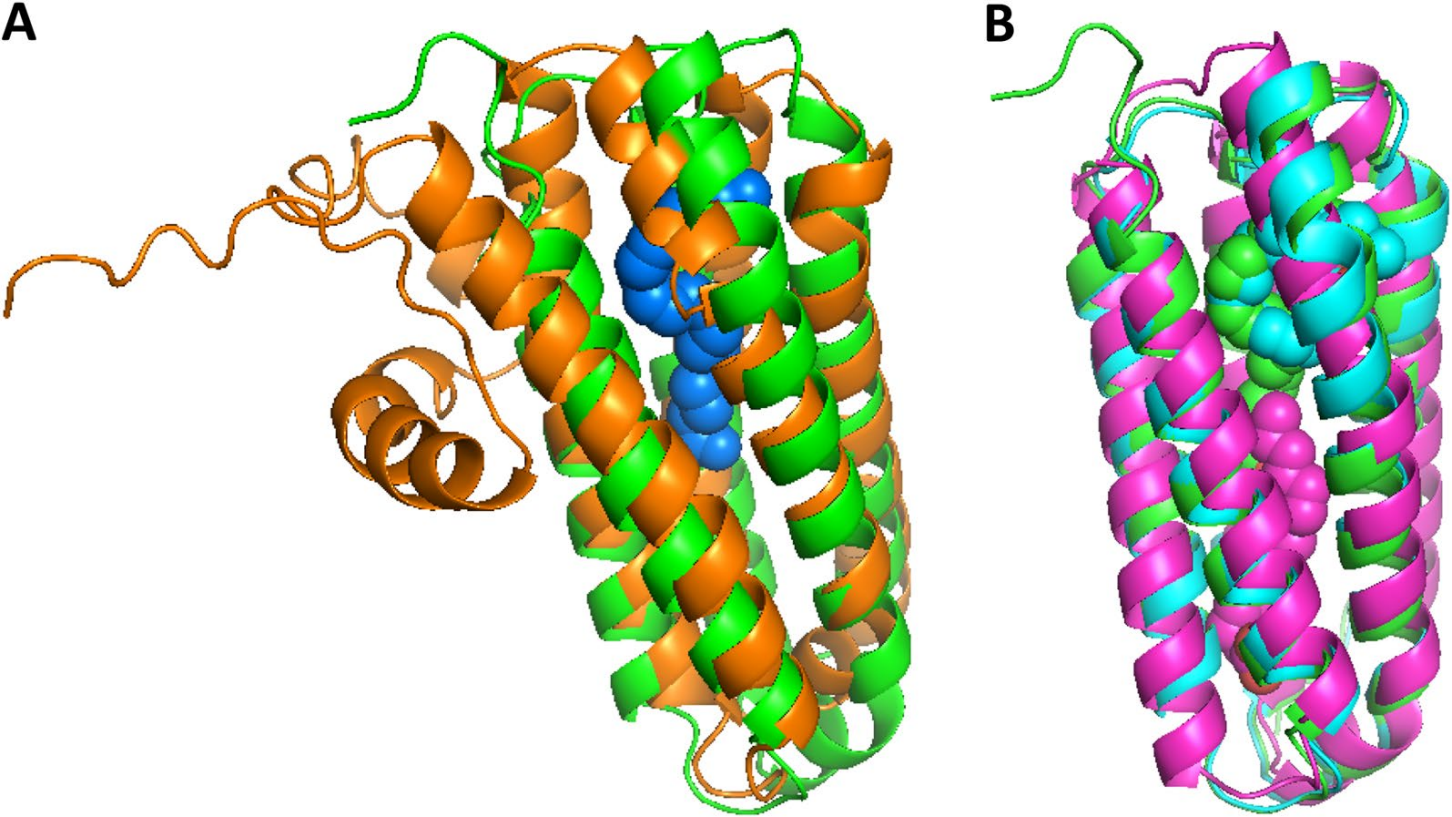
Superposition of the predicted structure of UP1 onto domain 2 of Mp1 in *Talaromyces marneffei*. **A** Left: Superposition of the predicted UP1 structure (orange) on Mp1 ligand-binding domain 2 (green), where a complex has been formed with arachidonic acids (blue spheres) (5CSD). **B** Right: Superposition of the structurally similar UP1 domains bound to one arachidonic acid

The three PDB structures indicated in bold are shown in Fig. 7

Remarkably, these structures are FA-binding domains and were found to bind to arachidonic acid (1 or 2 molecules) or palmitic acid [40]. A similar protein in apo form (i.e., without a bound FA chain) was previously observed in *Drechmania coniospora*. With the exception of the N-terminus extension, which seems to be specific to the *Y. lipolytica* UPs, all these proteins could be structurally similar, as their core domains display the same helice-bundle. As a result, they are highly likely to be functionally similar, as the topology of the helix bundle could lead to the comparable binding of FAs (Fig. 7). In the known complexes, FAs are always bound in an elongated hydrophobic pocket between the helices. Such a binding mode would also be possible in the predicted model for UP1 to UP4.

In the known complexes, the orientation of the bound FA and the position of its carboxylate group are variable. Indeed, in these FA-binding proteins, hydrogen bonds are established between the carboxylate group of either palmitic or arachidonic acid and the polar side chains of Q138 in domain 1 of Mp1 in *T. marneffei* (5E7X), of N105 and S165 in domain 1 of Mp1 in *T. marneffei* (5ECF chain B), and of S136 and S140 in domain 2 of Mp1 in *T. marneffei* (5FB7) (Table 5).

**Fig. 8:**
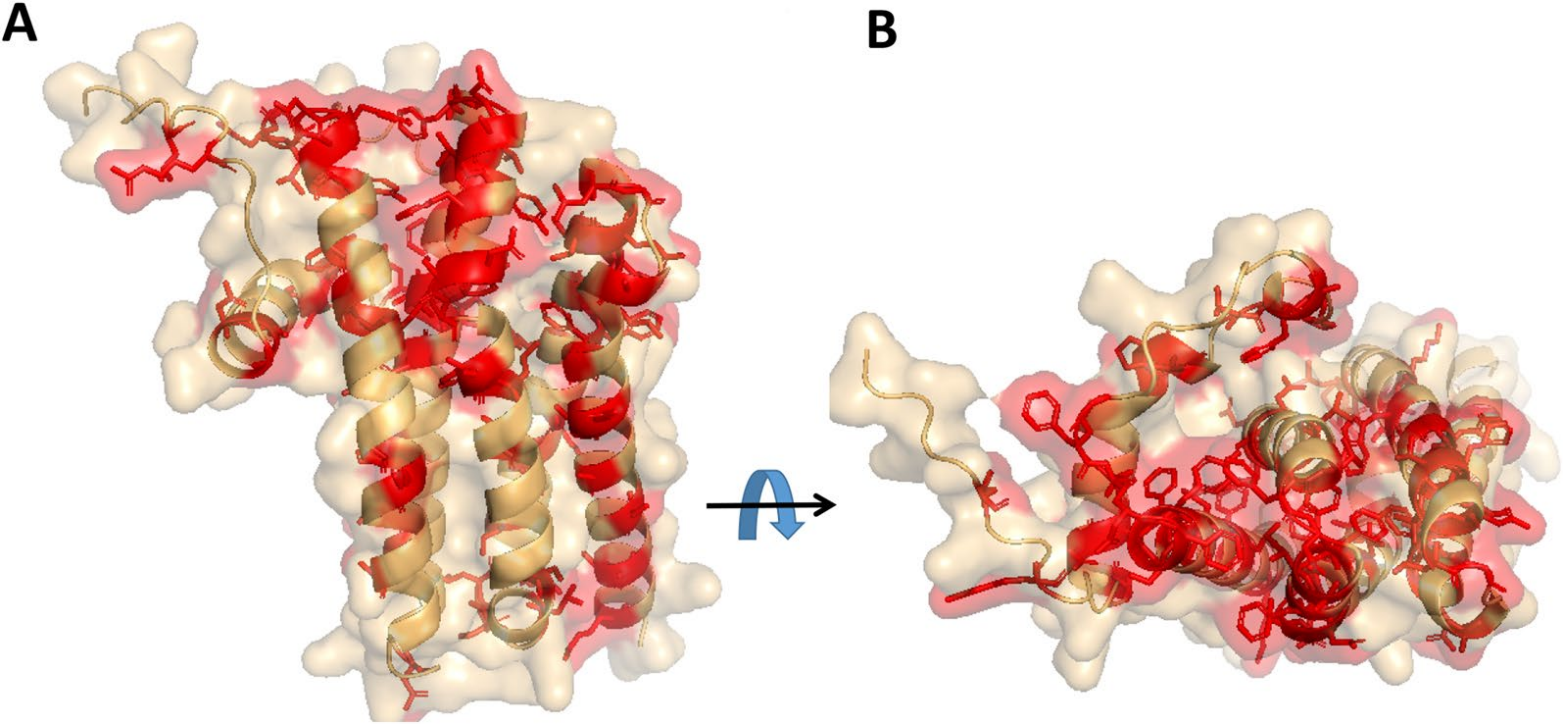
Predicted structure of UP1 to UP4. The conserved residues in UP1 to UP4 are shown using red sticks; they are projected onto the predicted structure of UP1 (shown in tan). **A** Left: The barrel is shown with helices 1, 3, and 5 running downward. **B** Right: Top view of barrel A. A set of conserved hydrophobic side chains are located on the surface of the helices oriented toward the inner cavity. Another set of conserved side chains forms a cluster on one extremity of the helical barrel close to the N-terminus extremities of helices 1 and 3. This pattern may suggest the existence of an interaction area for another, as-yet-unidentified biological partner

It was not possible to infer the retention of these carboxylate-binding positions in the UPs. Nevertheless, UP1 to UP4 should all have a putative binding site within the inner faces of the helices. The models display apolar residues, which are particularly well suited to binding with alkyl moieties (Fig. 8). Among them, L61, F101, F127, V131, W161, and F182 are strictly conserved in the four UPs (numbering: UP1 mature form). Another strictly preserved residue is Y116, which could possibly be a hydrogen-bonding partner for the FA’s carboxylate. To characterize FA binding capacity and specificity, it would be possible to carry out additional minimization to relax the structures and perform subsequent docking of FAs in UP1 to UP4, with either one or two bound molecules. However, such an exercise is beyond the scope of this paper.

## Discussion

In this study, we report the identification and preliminary characterization of a previously undescribed gene family with four members. The genes were identified during the data mining and analysis of the *Y. lipolytica* secretory proteome. The newly identified proteins encoded by these genes are highly similar to each other in terms of primary structure; hence, they are also highly likely to be related in function. No biological processes, cellular compartments, or molecular functions had been assigned to any of these proteins. The only indicator of function was the high-confidence prediction of signal peptides at the N-termini of the polypeptide chains, which suggests that all the proteins are secreted into extracellular space. When blasting the entire amino acid sequences for the UPs (working names: UP1 to UP4), no significantly homologous proteins were identified beyond this same group of proteins in different *Y. lipolytica* strains (i.e., no hits outside this group/species). Likewise, we were unsuccessful in discovering similar patterns via the identification of conserved motifs and subsequent screenings of databases, an approach that was expected to increase the probability of finding structural homologs. Based on these analyses, we concluded that this newly identified four-member protein family is unique to *Y. lipolytica*.

To reveal the biological function of UP1 to UP4, we generated a series of *Y. lipolytica* strains that overexpressed the UP-encoding genes individually in the JMY7126 background and in a quadruple-deletant (Q4) background (all four UP loci knocked out). The choice of the other characteristics of the host strain’s genetic background was based on the following criteria. First, for the analysis of strain growth and FA toxicity, we had to consider the solubility of the FAs in a water-based medium, the toxicity of the FAs to *Y. lipolytica* cells, and the mode of FA utilization [16, 28]. To solve the solubility problem, we used methyl ester derivatives. Furthermore, since short-chain FAs are toxic for *Y. lipolytica*, we used a strain that is devoid of the main secreted lipases, encoded by the *LIP2*, *LIP7*, and *LIP8* genes [10, 11] and hence decreased lipolytic activity. Second, to ensure controlled high-level overproduction of the UPs, we decided to use our erythritol-induced expression system, comprising a very strong hybrid-erythritol-induced promoter; the deletion of the *EYK1* gene in the host genome guarantees that erythritol is used for induction and not as a carbon source [34]. Indeed, as we have shown in our previous work, the erythritol-induced system is very efficient and convenient for the strictly controlled overproduction of proteins [34, 35, 41, 42]. The host strain JMY7126 bears the aforementioned lipase deletions, the main extracellular protease deletion (*XPR2*; advantageous for heterologous protein synthesis), and the *EYK1* gene deletion. We have also observed that heterologous protein production is improved in a strain with the *MHY1* gene deleted (to be published); this gene encodes a protein involved in hypha formation. Hence, *mhy1Δ* was introduced in the final step of strain construction (Fig. 3). Based on these requirements, we used the strain JMY7126 (*MATA ura3-302 leu2-270-LEU2-Zeta, xpr2-322, lip2Δ, lip7Δ, lip8Δ, lys5Δ, eyk1Δ*) in the construction of the multiple-deletant UP strains. This strain contains six background deletions; therefore, the terms “single” or “quadruple” deletion (Q1 to Q4) refer only to the UP genes. Considering that these background modifications are present in all the control, deletion, and overexpression strains, they should not impact the final interpretation of the results for the UPs.

As depicted in Fig. 2, the position of the intense bands in the profiles for the UP-overexpression strains under erythritol induction was unexpected. Considering the predicted molecular weight, the bands had lower molecular weights than expected. The proteomics analysis of the excised bands confirmed that, indeed, the UPs had exhibited unexpected migration patterns. In addition, the data indicated that the bands did not represent degradation products from the UPs, an inference based on the number of peptides identified and on the detection of peptides corresponding to the beginning and the end of the mature form. According to the structural conformations and protein structure analysis, the UPs are very small and compact proteins that can migrate through the polyacrylamide mesh faster than more expansive globular proteins. Hence, the unexpected migration pattern of the UPs may have resulted from their 3D conformation.

As none of the proteins in the databases exhibited similarity with the UPs, it was not possible to make any supported assertions about their putative function. However, knowing the biology, physiology, and genomic structure of *Y. lipolytica* as well as i) its marked ability to thrive in the presence of HSs (lipids, triglycerides, FAs) and ii) the growing number of multigene families involved in HS utilization, we hypothesized that the UPs might be involved in HS utilization. We therefore examined the growth of the constructed strains (Fig. 3) on media containing different FA methyl esters. The results of the drop tests (Fig. 4) suggested that, indeed, the UPs might be involved in the assimilation of HSs. Specifically, we observed that the Q4 strain was unable to grow in a minimal medium with methyl esters of C10 to C14. No such effect was seen when longer FAs were used. Strikingly, the overexpression of UP3 alleviated the growth limitations seen for Q4 on mC10, mC12, and mC14. The overexpression of UP2 and UP4 did the same for growth on mC12 and mC14.

Interpretation of the observed growth patterns was straightforward for mC12 and mC14. Complementation of the Q4 phenotype via the overexpression of one of the missing UPs enhanced the initially disrupted biological process of FA fixation and internalization. Hence, nontoxic FAs could be assimilated and metabolized by the growing cells. However, it has long been known that short-chain FAs, including C8 and C10, are not assimilated by transportation systems in *Y. lipolytica* [29] and that C8 is highly toxic to the cells [30]. Therefore, to gain more insight into why the growth of the Q4-UP3 strain was so efficient on mC10, we conducted an analysis of growth kinetics in the presence of C8 (Fig. 5). We observed that the quadruple-deletant was not subject to growth inhibition at 0.1–0.2% C8, as it grew better than the control strains with their basic constitutive levels of UP synthesis. Complementation with the UPs increased strain sensitivity. Such was particularly visible at 0.1% C8 with UP3 and UP4 overexpression and at 0.2% C8 with UP1 and UP4 overexpression, suggesting the proteins display specificity for this FA. Notably, the differences in strain sensitivity were concentration dependent; indeed, all the strains, irrespective of constructed genotype, were sensitive to C8 concentrations of > 0.3%. In the drop tests, conducted with an FA (C10–C18) concentration of 0.4%, severe limits on growth occurred for Q4 and its derivatives; the exception was Q4-UP3, which grew very well. Such observations are consistent with a previous study on FA internalization in *Y. lipolytica* [29], which clearly stated that the operation of the system is concentration dependent and exhibits specificity for the length of the aliphatic chain. Hence, it seems that UP3 enhances the toxicity of C8 at concentrations of > 0.1% via the protein’s enhanced delivery of the FA to the plasma membrane surface and subsequent flip-flop transportation. However, when binding to C10 present at a concentration of 0.4%, the protein reduces the FA’s availability in the medium. This hypothesis is supported by previous studies showing that the deletion of the *ABC1* gene, which encodes a protein involved in the exportation of alkanes, abolished growth on C10 alkanes, as it enhanced the toxic effect exerted by the compound [22]. Growth on mC10 is an equilibrium among i) the hydrolysis of the methyl ester (mC10) to form the free FA (C10), which is liberated by external lipases/esterases; ii) the fraction of the free FAs trapped by the UPs; and iii) the transported fraction that travels along, for example, the flip-flop/transporter pathways. Consequently, if the concentration of free FAs is too high (i.e., the activity of the extracellular lipases/esterases is too high), growth will be inhibited, as shown previously in a comparative analysis of growth and lipase production on mC10 for strains from the *Yarrowia* clade [8]. In contrast, low activity levels for the enzyme means limited liberation of the toxic free FAs; hence, cells can grow and metabolize FAs through the β-oxidation pathway. The phenotypes of the wild-type and mutant strains grown on both mC10 and C8 strongly support the idea that the UPs are involved in FA utilization.

**Fig. 9:**
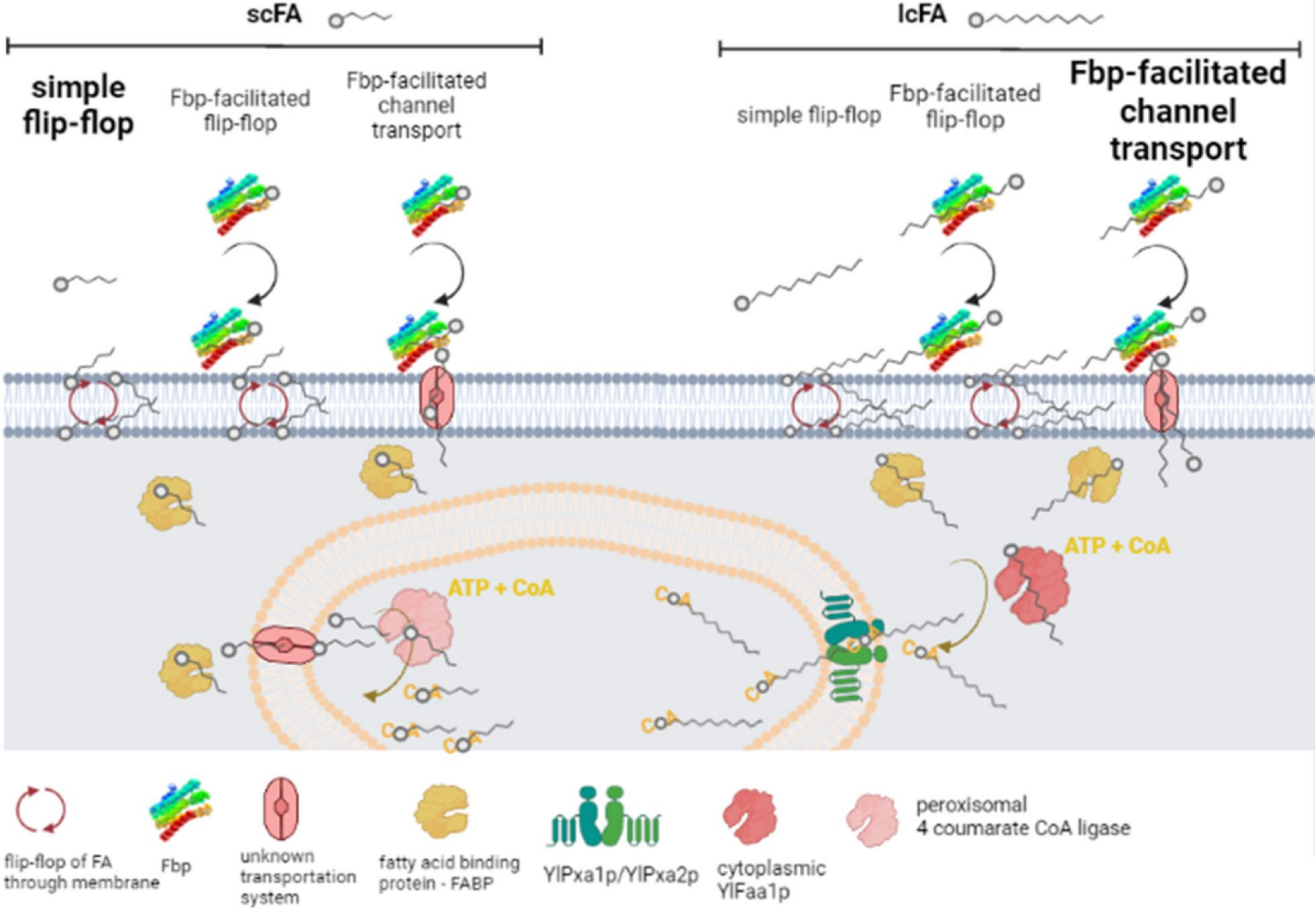
Proposed model of acyl-chain-length-dependent FA binding, transportation, and activation. The model is an updated version of a previous model for the transport and activation of FAs that was proposed by Dulermo and colleagues [16]. Depending on FA length, a different mode of fixation and transmembrane crossing predominates (indicated in bold). Abbreviations: scFA, short-chain fatty acid and lcFA, long-chain fatty acid. Simple flip-flop is the main mode of membrane crossing for scFAs. Fbp-facilitated channel transportation is the main mode for lcFAs. Irrespective of acyl chain length, after internalization, FAs are bound by intracellular FABPs (yellow/ochre). LcFAs are then activated by cytoplasmic YlFaa1p using ATP and CoA (dark pink) and transported into the peroxisome lumen (orange-pink bilayer) by YlPxa1p/YlPxa2p (blue green). ScFAs are first transported into the peroxisomes by an unknown transportation mechanism (light pink channel) and are activated once inside by peroxisomal 4 coumarate CoA ligase (light pink; inside the peroxisome). This diagram was created in BioRender (https://app.biorender.com/)

The sequence similarity between the UPs and other proteins was too low (sequence identity ranging between 7 and 11%) to identify structural analogs. However, the similarity of the structures predicted by the AlphaFold models was consistent and revealed accurate structural analogy with FA-binding proteins (Fig. 7). The UP structures are highly similar to each other, which was expected based on their primary structure similarity. Sequence conservation projected onto the predicted structure of the UPs indicated that the part of the barrel domain located near the N-terminus has a higher fraction of conserved side chains, which are also included at the N-terminus’ extremity. Such may point to a functional role and could suggest the presence of an interaction surface whose partners are as yet unidentified. The N-terminus of the sequence was trickiest to predict. It forms an extension stretching from the helical domain which is unique and of an unknown functional role. In the models, the core sequence is a helical barrel with a hydrophilic surface and a hydrophobic internal pocket that can bind to FAs. Most of the residues that shape the inner side are also strictly conserved from UP1 to UP4, which could guarantee a preserved binding mode. Accordingly, the ligand-binding domains of the protein Mp1, found to be structurally similar to the UPs, are known to be virulence factors. They trap the proinflammatory lipid mediator arachidonic acid, for which they have a high affinity, and consequently alter the host response to infections [40, 43]. In *Y. lipolytica*, which is a nonpathogenic yeast species [44], such biological processes seem to be irrelevant. Nevertheless, the molecular function of aliphatic chain recognition appears to be both useful and relevant in biological processes such as FA transportation or internalization. Additionally, this finding is particularly striking in *Y. lipolytica*’s metabolic context, especially because no such molecules have been described previously. Also, redundancy among the four highly similar UPs cannot be ruled out. It emphasizes both the expected fine tuning of specificity for distinct FAs and, possibly, the importance of these proteins in *Y. lipolytica*’s life cycle. Altogether, this discovery fills a substantial gap in knowledge and needs to be more precisely deciphered in short order. Based on these results, we have concluded that the UPs are involved in the binding of free FAs in the medium and in their delivery to the cell surface, as described in the proposed model (Fig. 9).

Understanding how FAs can enter cells is of great interest. Until now, no extracellular proteins able to bind to FAs had been identified. Here, we highlight a newly discovered multigene family present only in *Y. lipolytica*. After binding to these secretory proteins, the FAs are solubilized, sequestered, and transported into the cells.

Further research is needed to determine the binding specificity of these eFbp. For example, in vitro binding tests could explore the specificity of these proteins for different types of FAs, such as polyunsaturated FAs, hydroxylated FAs, and fatty alcohols. Another challenge is to identify the role of the N-terminus, which seems to be “floppy.” It may be involved in the docking of the protein to the cell surface; in the binding of the protein to a transport channel for a Fbp-facilitated flip-flop; or in a Fbp-facilitated channel transport mechanism, depending on FA chain length.

## Conclusions

The phenotypes of the eFbp mutants and the eFbp-overexpression strains when grown on HSs strongly support the idea that UP1 to UP4 are FA-binding proteins. The structures predicted using a deep learning procedure, in association with the systematic structure comparisons, further support that this unique protein family is involved in FA binding, solubilization, sequestration, and, likely, transport into cells. It is expected that these proteins may have relevant applications in lipid biotechnology, such as improving FA secretion/production in yeasts and reducing the toxicity of strains that secrete short-chain FAs. These proteins could also be relevant in improving the bioconversion of FAs, such as the bioconversion of linoleic acids into conjugated linoleic acid.

## Materials and methods

### Strains and cloning strategy

The first set of yeast strains used in this study was constructed in the background of the *Y. lipolytica* JMY7126 host strain, developed previously [34, 35]. This strain is unable to utilize erythritol (ERY; *Δeyk1*), which, in combination with the ERY-inducible promoter, makes it an efficient host for inducible overproduction of cloned genes. Routine cultivation was conducted according to standard protocols [3]. The second set of yeast strains was constructed via successive gene deletion, resulting in the quadruple-deletant JMY8761 (Q4). The Q4 strain was used as the background in the construction of the strains that overexpressed the UPs individually, eliminating the interference introduced by native constitutive UP expression. All the strains used in this study are listed in Table 3.

Vector construction and subcloning were conducted using the *Escherichia coli* DH5alpha strain, which was routinely maintained according to standard protocols [45]. All the oligonucleotides used for cloning are listed in Additional file 1: Table S1. The plasmids are listed in Table 3.

The cloning procedures followed standard molecular biology protocols [45]. Restriction enzymes and T4/T7 DNA ligases were obtained from New England Biolabs (MA, USA). PCR amplification was performed using an Applied Biosystems 2720 Thermal Cycler and either GoTaq DNA polymerases (Promega, WI, USA) or Q5 High-Fidelity DNA Polymerase (New England Biolabs). The PCR fragments were purified using a QIAgen Purification Kit (Qiagen, Hilden, Germany), and plasmid DNA was isolated using a QIAprep Spin Miniprep Kit (Qiagen). The four target genes were amplified from the *Y. lipolytica* genomic DNA template and cloned into the JMP4230 vector using BamHI/AvrII restriction digestion. The destination vector, JMP4230, is a variant of the JMP62 shuttle vector series [46], bearing the strong ERY-inducible promoter pHU8EYK1 [42], the tLip2 terminator, the excisable *URA3*ex auxotrophy selection marker, and the zeta integration sites that flank the expression cassette (Additional file 1: Fig. S1). Gene expression cassettes were obtained by NotI digestion of the corresponding plasmid and used to transform *Y. lipolytica* strains via the lithium acetate method, as described previously [3]. Two positive subclones, bearing one of the four JMP62-based overexpression cassettes, were stored for further research.

The quadruple-deletion mutant (Q4), in which all four genes of interest were deleted, was generated using the CRISPR-Cas9 system, as described previously [36]. Proper gene deletion was verified via colony PCR and by identifying the strains that contained the expected deletion between the two guides. The expected PCR products were 1128/615, 1381/939, 999/4643, and 1005/457 (fragment size in bp in the wild type/fragment size in bp in the deletion mutant) for UP1 to UP4, respectively. Proper genomic integration of the JMP62-based overexpression cassettes and gene disruption were verified via PCR and sequencing.

### Overexpression of UPs

To investigate the expression levels of the four target proteins, the overexpression strains were grown in liquid, batch cultures in YNBD2 (noninduced medium) and in YNBD2E (erythritol-induced medium) at 28 °C and 150 rpm for 48 h. The minimal YNB medium contained 0.17% (w/v) yeast nitrogen base (without amino acids and ammonium sulfate, YNBww), 0.5% (w/v) NH_4_Cl, and 0.2% (w/v) glucose; it was supplemented with 0.5% (w/v) erythritol for induction. The media were buffered with 50 mM phosphate buffer at pH 6.8. Samples were collected at 48 h; biomass was separated by centrifugation (5000 *g* for 5 min); and the supernatants were used in further analyses.

### Drop tests on agar plates

Precultures were grown overnight (180 rpm, 28 °C) in YPD. The cells were centrifuged, washed with YNB, and resuspended at an OD600 of 1. Successive tenfold dilutions were performed (10^0^–10^–4^), and 10 µl of each dilution was spotted onto YNB plates containing the indicated FAs and lipids. The following FAs were used in our study: mC10, methyl decanoate (SAFC, 99%); mC12, methyl laurate (Sigma Aldrich, 98%); mC14, methyl myristate (SAFC, 98%); mC16, methyl palmitate (SAFC, 97%); tributyrin (ACROS, 98%); triolein (Fluka, 65%); and C8, octanoic acid (Aldrich, 98%). The minimal YNB medium contained 0.17% (w/v) yeast nitrogen base (without amino acids and ammonium sulfate, YNBww), 0.5% (w/v) NH_4_Cl, and 0.2% (w/v) glucose; it was supplemented with 0.5% (w/v) erythritol for induction. To complement strain auxotrophy, uracil (0.1 g/L) and lysine (0.2 g/L) were added as required. The media were buffered with 50 mM phosphate buffer at pH 6.8. Stock solutions of the methyl esters of the FAs and of the lipids were subjected to sonication three times for 1 min in the presence of Tween 40 (Sigma) and used at a final concentration of 0.4%. Solid media were created by adding 1.6% agar. The plates were incubated at 28 °C. The drop tests were conducted twice, with two subclones of each specific genotype. Pictures were taken every 24 h. Representative images are shown.

### Growth in microplates

In this experiment, we used two control strains: (i) a JMY7126 derivative (JMY8651; control) bearing the *mhy1*Δ deletion and (ii) a Q4 derivative in which *MHY1* had been deleted (JMY8777; Q4). All the strains contained the same auxotrophy (Table 3). Overnight precultures in YPD were centrifuged and washed with YNB. The cell suspensions were standardized to an OD600 of 0.1. The yeast strains were grown in 96-well plates in 200 µL of minimal YNB medium containing glucose (2 g/L) and different concentrations of FAs. The media were supplemented with erythritol (5 g/L) under induction conditions and with ethanol (8 g/L) under control conditions (i.e., without any FAs). An ethanol solution of octanoic acid (C8) was added to the medium to achieve a final concentration of 0.1% to 0.2% of C8. The cultures were maintained at 28 °C under constant agitation using a Biotek Synergy MX Microplate Reader (Biotek Instruments, Colmar, France). Growth was followed by measuring the culture’s optical density at 600 nm every 30 min for 72 h.

### SDS–PAGE and identification of polypeptides via mass spectrometry

Samples were taken from the shake flask cultures, grown either with or without induction with erythritol. They were concentrated tenfold on an Amicon Ultracel-10 Membrane (Millipore, Molsheim, France) and subjected to SDS–PAGE, according to a standard methodology [45, 47]. The concentrated supernatants were denatured by boiling (5 min with Laemmli buffer) and then resolved using gradient SDS–PAGE Novex 4–12% in Tris–glycine buffer (Life Technologies). The molecular mass (MM) standard was SeeBlue^®^ Plus2 Pre-Stained Standard (Thermo Fisher Scientific, Villebon sur Yvette, France), which contains standard proteins ranging from 3 to 198 kDa.

Bands displaying approximately the expected size were excised from the gels. They were washed twice with 30 µL of 50% acetonitrile (ACN)/50 mM ammonium bicarbonate (NH_4_HCO_3_); they were then dehydrated with 30 µL of ACN. The disulfide bonds were reduced via exposure to 100 mM dithiothreitol (DTT, Sigma) for 30 min at 56 °C. Cysteine residues were alkylated via incubation with 50 mM iodoacetamide for 45 min in darkness at room temperature. Digestion was carried out overnight at 37 °C with 10 ng of trypsin (Promega). The peptides were extracted using 30 µL of 40% ACN/0.1% trifluoroacetic acid (TFA), followed by a treatment with 30 µL of ACN. The samples were vacuum dried (SpeedVac, Savant™ SPD121D, Thermo Fisher Scientific), suspended in 20 µL of loading buffer (2% ACN/0.1% TFA), and subject to LC–MS/MS.

Mass spectrometry was performed at the PAPPSO platform (MICALIS, INRAE, Jouy-en-Josas, France; http://pappso.inrae.fr/) using an Orbitrap Discovery (Thermo Fisher Scientific) coupled to an UltiMate™ 3000 RSLCnano System (Thermo Fisher Scientific, San José, USA). A 4-μL treated sample was loaded at 20 μL/min on a precolumn (µ-Precolumn, 300 µm i.d × 5 mm, C18 PepMap100, 5 µm, 100 Å, Thermo Fisher Scientific). After 3 min, the precolumn cartridge was connected to the separating column Acclaim PepMap RSLC nanoViper (C18 particle size = 3 µm, 500 mm in length, 75 µm i.d., Thermo Fisher Scientific). The loading buffer was 2% ACN/0.1% TFA; resolution buffer A was 0.1% AF/98% H_2_O; and resolution buffer B was 0.1% AF/80% ACN. The runs were executed at 300 nl/min with a linear gradient from 0 to 35% of buffer B for 25 min, including regeneration (98% of buffer B). One run took 54 min. Data-dependent acquisition in Top 8 was achieved with CID collision mode.

MS Data Analyses. The four UP sequences for *Y. lipolytica* were added to a bovine and a contaminant database (keratins). Protein identification was performed as described previously, using X!TandemPipeline v. 0.2.10, run with a precursor mass tolerance of 10 ppm and a fragment mass tolerance of 0.5 Da [48]. Enzymatic cleavage rules were set to trypsin digestion (after Arg and Lys, unless Pro immediately follows); no semi-enzymatic cleavage rules were allowed. The fixed modification was set to cysteine carbamidomethylation and methionine oxidation, which were considered to be potential modifications. The identified proteins were filtered as follows: (1) peptide E-value < 10^–2^ with a minimum of 2 peptides per protein and (2) a protein E-value of < 10^–4^.

### Bioinformatics

#### BLAST—database search for similar sequences and signal peptide prediction

First, the fasta sequences of UP1, UP2, UP3, and UP4 from *Y. lipolytica* strain CLIB122 were retrieved from GRYC (http://gryc.inra.fr/) and analyzed using the Phobius tool from EBI, which predicts transmembrane topology (https://www.ebi.ac.uk/Tools/pfa/phobius/). Secretory potential as well as the primary and secondary amino acid structure of the signal peptides (SPs) were predicted using the SignalP [49], TargetP [50], and PrediSi [51] tools.

Then, the sequences were aligned using the ClustalW algorithm because of its accuracy and precision (with default parameters) [52]. The following motifs were identified as conserved: AAP[TS], GT[KR]FD[KQ]AVY[EA]F[IL][VI]NSGx[KS]DFL, GFLDFSGY, [IF]LxSPLL, [TS]GF[DN]RA, W[IL]FGxKQTVQ, and APV[FY][TS]LAPxxFA; the residues in brackets are allowed to experience substitutions. The UniProt database was screened with the EMBOSS Fuzzpro tool to extensively search for sequences that could contain a combination of these motifs to identify putative homologs. In parallel, to obtain functional information, the sequence proteins were submitted and scanned against the PROSITE collection of motifs using the Expasy/PROSITE webserver (https://prosite.expasy.org/scanprosite/).

#### BLAST and 3D structure analysis

HHpred was used to search for structural homologies in the PDB [53]. Since none could be retrieved from the PDB at this stage, we used AlphaFold structure predictions, obtained via artificial intelligence [38]. The sequence of *YALI0D03245g1* (UP1) was submitted to AlphaFold2 using the ColabFold server [54]. The 3D structure predicted by AlphaFold was then compared to all known protein structures using Dali [39].

### Statistical analysis

Statistical analysis of the final time-point data on growth in C8 0.2% liquid media was performed in RStudio version 2022.07.1 (R version 4.2.1) [55]. A post-hoc Tukey HSD test from the agricolae package (version 1.3–5) was used [56].

## Supplementary Information

**Additional file 1:** **Figure S1.** Schematic representation of the destination vector JMP4230, a variant of JMP62 shuttle vector series, bearing the strong Erythritol-inducible promoter pHU8EYK1, tLip2 terminator, the excisable URA3ex auxotrophy selection marker, and zeta integration sites, flanking the expression cassette. Bacterial part bearing ori and kanamycin resistance marker was removed, prior to the yeast transformation, by NotI restriction enzyme digestion. Main unique restriction sites are indicated; ClaI-BamH1 for promotor exchange, BamH1-AvrII for gene cloning and I-SceI for marker exchange. **Figure S2.** The rank 1 to rank 5 models of UP1 structure independently predicted by AlphaFold were superimposed. Each chain is colored in blue to red from N-end to C end. The five long helices (residues 50-200 in matured protein) are consistently predicted in the same relative positions. The predicted structure of residue 1 to 50 are more variable between predictions. **Table S1.** List of primers used in this study.

## Data Availability

All data generated or analyzed during this study are included in this published article and its additional information files.
