# Supplementary material for: A unique, newly discovered four-member protein family involved in extracellular fatty acid binding in Yarrowia lipolytica
Source: Microb Cell Fact. 2022 Oct 1;21:200. doi: 10.1186/s12934-022-01925-y (PMC9526294; doi:10.1186/s12934-022-01925-y)
Supplement: Supplementary file 1 — Additional file 1: Figure S1. Schematic representation of the destination vector JMP4230, a variant of JMP62 shuttle vector series, bearing the strong Erythritol-inducible promoter pHU8EYK1, tLip2 terminator, the excisable URA3ex auxotrophy selection marker, and zeta integration sites, flanking the expression cassette. Bacterial part bearing ori and kanamycin resistance marker was removed, prior to the yeast transformation, by NotI restriction enzyme digestion. Main unique restriction sites are indicated; ClaI-BamH1 for promotor exchange, BamH1-AvrII for gene cloning and I-SceI for marker exchange. Figure S2. The rank 1 to rank 5 models of UP1 structure independently predicted by AlphaFold were superimposed. Each chain is colored in blue to red from N-end to C end. The five long helices (residues 50-200 in matured protein) are consistently predicted in the same relative positions. The predicted structure of residue 1 to 50 are more variable between predictions. Table S1. List of primers used in this study. [file 12934_2022_1925_MOESM1_ESM.docx]

Additional file 1

**A unique, newly discovered four-member protein family involved in extracellular fatty acid binding in Yarrowia lipolytica**

Djamila Onésime^1^, Léa Vidal^1^, Stéphane Thomas^1^, Céline Henry^2^, Véronique Martin^3^, Gwenaëlle André^3^, Piotr Kubiak^4^, Philippe Minard^5^, Ewelina Celinska^4^, Jean-Marc Nicaud^1ǂ^

^1^Micalis Institute, INRAE, AgroParisTech, Université Paris-Saclay, 78350 Jouy-en-Josas, France

^2^Plateforme d’Analyse Protéomique Paris Sud-Ouest (PAPPSO), INRAE, MICALIS Institute, Université Paris-Saclay, 78350 Jouy-en-Josas, France.

^3^Université Paris-Saclay, INRAE, MaIAGE, 78350, Jouy-en-Josas, France

^4^Department of Biotechnology and Food Microbiology, Poznan University of Life Sciences, ul. Wojska Polskiego 48, 60-627 Poznań, Poland

^5^Université Paris-Saclay, CEA, CNRS, Institute for Integrative Biology of the Cell (I2BC), 91198, Gif-sur-Yvette, France

^ǂ^Corresponding author: Micalis Institute, INRAE-AgroParisTech, UMR1319, Team BIMLip: Integrative Metabolism of Microbial Lipids, domaine de Vilvert, 78352 Jouy-en-Josas, France

Tel: +33 1 74 07 18 20

E-mail : [jean-marc.nicaud@inrae.fr](mailto:jean-marc.nicaud@inrae.fr)

**Keywords**: *Yarrowia lipolytica*, lipid, fatty acid binding protein, fatty acid transport, secretion

**Additional Figure S1**


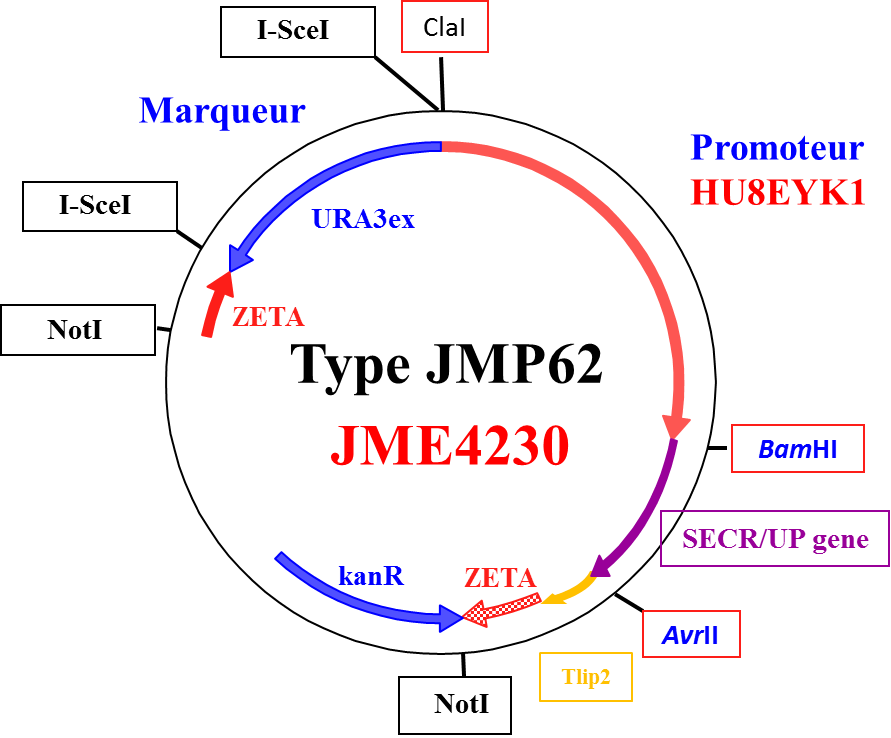


***Additional Figure S1****. Schematic representation of the destination vector JMP4230, a variant of JMP62 shuttle vector series, bearing the strong Erythritol-inducible promoter pHU8EYK1, tLip2 terminator, the excisable URA3ex auxotrophy selection marker, and zeta integration sites, flanking the expression cassette. Bacterial part bearing ori and kanamycin resistance marker was removed, prior to the yeast transformation, by NotI restriction enzyme digestion. Main unique restriction sites are indicated; ClaI-BamH1 for promotor exchange, BamH1-AvrII for gene cloning and I-SceI for marker exchange.*

**Additional Figure S2**


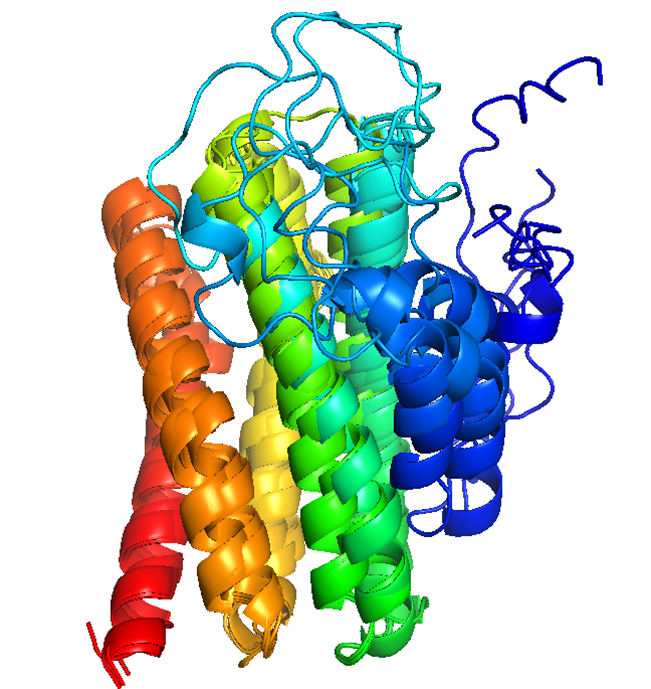


***Additional Figure S2****. The rank 1 to rank 5 models of UP1 structure independently predicted by AlphaFold were superimposed. Each chain is colored in blue to red from N-end to C end. The five long helices (residues 50-200 in matured protein) are consistently predicted in the same relative positions. The predicted structure of residue 1 to 50 are more variable between predictions.*

**Additional Table S1**

***Additional Table S1 List of primers used in this study***

F/fw: forward primer; R/rev: revers primer, G.OE.: Gene overexpression; BamH1 and AvrII sites are bolded and underlined.

| **Gene** | **Primer name** | **Primer sequence** | **use** |
| --- | --- | --- | --- |
| YALI0D03245g | ST081-prot3245A-F | ATT**GGATCC**CACAATGAAGTTCTCTCACGTCACC | G.OE. |
|  | ST082-prot3245A-R | TTA**CCTAGG**TTAGTTACCAAGCTTCTTGGTG | G.OE. |
| YALI0F04598g | ST083-prot4598B-F | ATT**GGATCC**CACAATGCAGTTCTCCACTCTTGCC | G.OE. |
|  | ST084-prot4598B-R | TTA**CCTAGG**TTAGTGTCCTCCAGCCTTCTTC | G.OE. |
| YALI0C05687g | ST085-prot4687C-F | ATT**GGATCC**CACAATGAAGTTCTCCGCCGTTGC | G.OE. |
|  | ST086-prot4687C-R | TTA**CCTAGG**TTAGTTGTTCTTCTGGCCATCG | G.OE. |
| YALI0F04620g | ST087-prot4620D-F | ATT**GGATCC**CACAATGAAGCTCTCTGCCGTCAC | G.OE. |
|  | ST088-prot4620D-R | TTA**CCTAGG**CTAGTTGCTACCGTTCTTGATC | G.OE. |
|  |  |  |  |
| YALI0C05687g | UP1_682fw | TTTTCACTTTCCCCCCCCTC | Verification |
| YALI0C05687g | UP1_1686rev | AACATGGCAGCATGGCAAC | Verification |
| YALI0D03245g | UP2_549Fw | ACATCACCACATCCCACACC | Verification |
| YALI0D03245g | UP2_1676Rev | ACCTCTGAAACCAACTGAAAC | Verification |
| YALI0F04598g | UP3_478fw | AACCTCACACCACAACCAC | Verification |
| YALI0F04598g | UP3_1858rev | AAACATCTCTTTCTCGCCATC | Verification |
| YALI0F04620g | UP4_689fw | TCAAACCCTATCTATCGTCCTC | Verification |
| YALI0F04620g | UP4_1687rev | ACTCAAGTCCCATCTACAGTC | Verification |
| YALI0B21582g | SeqFila-fwd | CATATCCCTCTCTCCAGC | Verification |
| YALI0B21582g | SeqFila-rev | CTTACAAAGTGGACAAGCG | Verification |
|  | | | |
| YALI0C05687g | UP1_Seq | CTCGTTAATCTCGTCTCCAATG | sequencing |
| YALI0D03245g | UP2_Seq | TTCTCTCACTCACCAAGCTTC | sequencing |
| YALI0F04598g | UP3_seq | CACCGCAACACCCTCCATAATG | sequencing |
| YALI0F04620 | UP4_seq | GCCGTCACTTTCATTGCTCTC | sequencing |
| YALI0B21582g | Fila-intern-verif-fw-2 | GCATCGCATCTAATCAGG | sequencing |
| YALI0B21582g | Fila-intern-verif-Rev | GTCGGAGCACGAAGAGGTGTCATGC | sequencing |
| sgRNA target sequence | | | |
| YALI0C05687g | C05687g_gRNA69rv | AAACAAGAATCTCCCGGGAC | gRNA |
| YALI0C05687g | C05687g_gRNA620fw | AGCTATGCTCGCCGTCCAGT | gRNA |
| YALI0D03245g | D03245g_gRNA55rv | CGGGACTCCAGCTGCTGGGT | gRNA |
| YALI0D03245g | D03245g_gRNA567rv | TAACGTCAGCGGCAGAGCCC | gRNA |
| YALI0F04598g | F04598g_gRNA55rv | CGAGCAACCAGAGAGTTGGT | gRNA |
| YALI0F04598g | F04598g_gRNA500fw | CCTCGAGTCTCCCCTTCTCC | gRNA |
| YALI0F04620g | F04620g_gRNA77rv | GTAGAGAGTGTTGTCTTCCC | gRNA |
| YALI0F04620g | F04620g_gRNA612rv_F | AGTTGATGGCGAGTTGGGCT | gRNA |
| YALI0B21582g | B21582g_gRNA | GGCGACAGCATGTAAATGGG | gRNA |
